# DNA image cytometry of bronchial washing as a diagnostic adjunct to radial endobronchial ultrasound‐guided sampling of peripheral lung lesions: A single center prospective study

**DOI:** 10.1111/crj.13703

**Published:** 2023-12-11

**Authors:** Cuiyan Guo, Qi Zhang, Yan Hu, Junfang Huang, Shixuan Wang, Guangfa Wang

**Affiliations:** ^1^ Department of Respiratory and Critical Care Medicine Peking University First Hospital Beijing China

**Keywords:** DNA aneuploidy, DNA image cytometry, lung cancer diagnosis, peripheral lung lesions, radial endobronchial ultrasound

## Abstract

**Objective:**

The objective of this study is to study the adjunct role of combining DNA aneuploidy analysis with radial endobronchial ultrasound (R‐EBUS)‐guided sampling for diagnosis of peripheral lung lesions (PPLs).

**Method:**

A single‐center prospective study was conducted in patients undergoing R‐EBUS‐guided sampling for PPLs. DNA image cytometry (DNA‐ICM) was used to analyze DNA aneuploidy in bronchial washing from the bronchial segment of the PPL. Clinical information, R‐EBUS data, pathology, DNA‐ICM results, and follow‐up data were analyzed. Sensitivity, specificity, and predictive values for R‐EBUS‐guided sampling, DNA‐ICM, and the two methods combined were measured. Binary logistic regression was performed to determine influencing factors on diagnostic positivity rate. Receiver operating characteristic (ROC) curve analysis was used to determine the optimal cutoff point for DNA‐ICM.

**Results:**

A total of 101 patients were enrolled. Sixty‐four (63.4%) patients had confirmed malignant tumor, of whom 33 were confirmed by R‐EBUS‐guided sampling (biopsy and/or bronchial brush and wash cytology), and 31 by surgery or percutaneous lung biopsy. Thirty‐seven patients were finally considered to have benign lesions, based on clinical information and 1‐year follow‐up. The sensitivity for malignant disease was 51.6% by R‐EBUS, and specificity was 100%. DNA‐ICM had a sensitivity of 67.2% and a specificity of 86.5%. When combining the two methods, sensitivity increased to 78.1% and specificity was 86.5%. Lesion size and whether the R‐EBUS probe was located in the lesion were significantly associated with positivity rate of the combined methods. The optimal cutoff point for DNA‐ICM was 5c for max DNA content, and 1 for aneuploid cell count (sensitivity 67.2%, specificity 86.5%, accuracy 63.4%).

**Conclusion:**

In malignant PPLs, DNA‐ICM combined with R‐EBUS‐guided sampling can improve diagnostic positivity compared with either method alone.

## Graphical abstract

This study shows that the combined use of DNA‐ICM can improve diagnostic yield when using R‐EBUS to sample a PPL. Based on the study results, we suggest the joint use of DNA‐ICM with R‐EBUS‐guided sampling for PPLs. Closer follow‐up or alternative methods of biopsy are recommended for cases with negative pathology but positive DNA‐ICM.

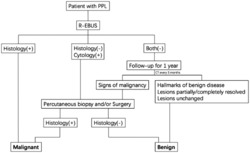

## 1 INTRODUCTION

Lung cancer is the leading cause of cancer mortality worldwide.^1^ The extensive use of low‐dose CT has detected a vast number of peripheral lung lesions (PPLs), but definitive diagnosis cannot be made without biopsy.

Sensitive and safe methodology remains challenging to clinicians despite the abundance of methods developed. Percutaneous lung biopsy has a high yield of over 90% but also high risks.^2^, ^3^ Pneumothorax and bleeding incidences could be as high as 10%–40% and 26%–33%, respectively.^3^ Severe complications such as air embolism and tumor implantation can occur.^4^, ^5^, ^6^ Radial endobronchial ultrasound (R‐EBUS) has improved the diagnostic yield of PPLs significantly, albeit far from perfect, and requires simultaneous use of other technologies, such as virtual bronchoscopy, electromagnetic navigation, bronchoscopic transparenchymal nodule access, or ultrathin bronchoscopy, with reported yields ranging between 69%–90.3%.^2^, ^7^, ^8^, ^9^ R‐EBUS in combination with rapid on‐site evaluation could significantly improve the diagnostic efficacy of PPLs.^10^ R‐EBUS has fewer complications, with reported incidence of pneumothorax at 1.5%^9^ but has lower diagnostic yield than percutaneous procedures, leaving room for improvement in more sensitive and safer methods.

Tissue biopsy is pivotal for cancer diagnosis. However, bronchoscopic assessment of PPLs may be limited by airway accessibility, requiring different approaches. Liquid biopsy has been used in evaluating suspected lung cancer in blood plasma. Fluid from lung segments or subsegments can be easily harvested by bronchoscopy. It can be rationally speculated that the combination of bronchoscopic liquid biopsy may increase the diagnostic yield of R‐EBUS‐guided sampling for PPLs.

Computer‐assisted DNA image cytometry (DNA‐ICM) is a fully automated DNA abnormality analysis for early detection of cancer.^11^ Although DNA‐ICM is widely used in cancer screening and diagnosis, there are few studies in the field of lung cancer, particularly PPLs. Xing et al. found that sputum DNA‐ICM may be a better alternative for cancer detection compared with conventional cytology.^12^ However, other studies showed controversial results.^13^, ^14^ Xing's study demonstrated that squamous cell lung cancer and late‐stage adenocarcinoma have 100% sensitivity with automated sputum cytology but only 25% in stage I adenocarcinoma.^12^ The main issue is that sputum is from the pan‐respiratory tract rather than any localized segment and can submerge localized cytology information and reduce the sensitivity of early lung cancer detection, while bronchial washing from the segment of the PPL is a more targeted approach. Therefore, combining localized bronchial sampling and DNA‐ICM may increase the diagnostic yield of early lung cancer.

A previous study in our center by Hu et al. combined DNA‐ICM with cytology in analysis of bronchial washings from 626 patients and found significantly increased sensitivity of the combined methods for diagnosis of lung cancer compared to cytology alone (75.8% vs. 53.3%, *P* < 0.05).^13^ Guo et al. retrospectively explored the diagnostic value of DNA‐ICM combined with R‐EBUS‐guided sampling in 42 patients with PPLs and demonstrated a trend in increased sensitivity with the combined methods.^15^ These preliminary studies showed promise for combining DNA‐ICM to increase the diagnostic yield of R‐EBUS‐guided sampling for PPLs, necessitating further prospective studies.

Herein, we conducted a prospective study to verify whether DNA‐ICM of bronchial washing could perform an adjunct role in the diagnosis of PPLs with radial EBUS‐guided sampling.

## 2 METHODS

### 2.1 Study design

This study was a single‐center prospective study approved by the Ethics Committee of Peking University First Hospital (2019‐072) and was conducted between March 2019 and September 2021. Patients scheduled to undergo R‐EBUS for PPLs were consecutively screened and enrolled by the following: Inclusion criteria: (a) patients with PPLs on CT and scheduled for R‐EBUS, (b) age equal or greater than 18 years, (c) signed informed consent; exclusion criteria: (a) unable to adhere to the follow‐up schedule, (b) contraindicated to bronchoscopy, (c) life expectancy <1 year, (d) lesions visible with white light bronchoscopy, (e) lesions undetectable by radial endobronchial ultrasonography, (f) clinical manifestations of infection or purulent bronchial secretion under bronchoscopy, (g) cell number of the specimen was insufficient for analysis (<500 cells).

Assuming prevalence of 60%, sensitivity of R‐EBUS‐guided sampling of 65%, and sensitivity of combined methods of 90%, a minimum of 69 patients would provide 80% power and a two‐sided significance level of 0.05 to detect the sensitivity. A final sample size of 82 patients was calculated after adjusting for anticipated dropouts.

**FIGURE 1:**
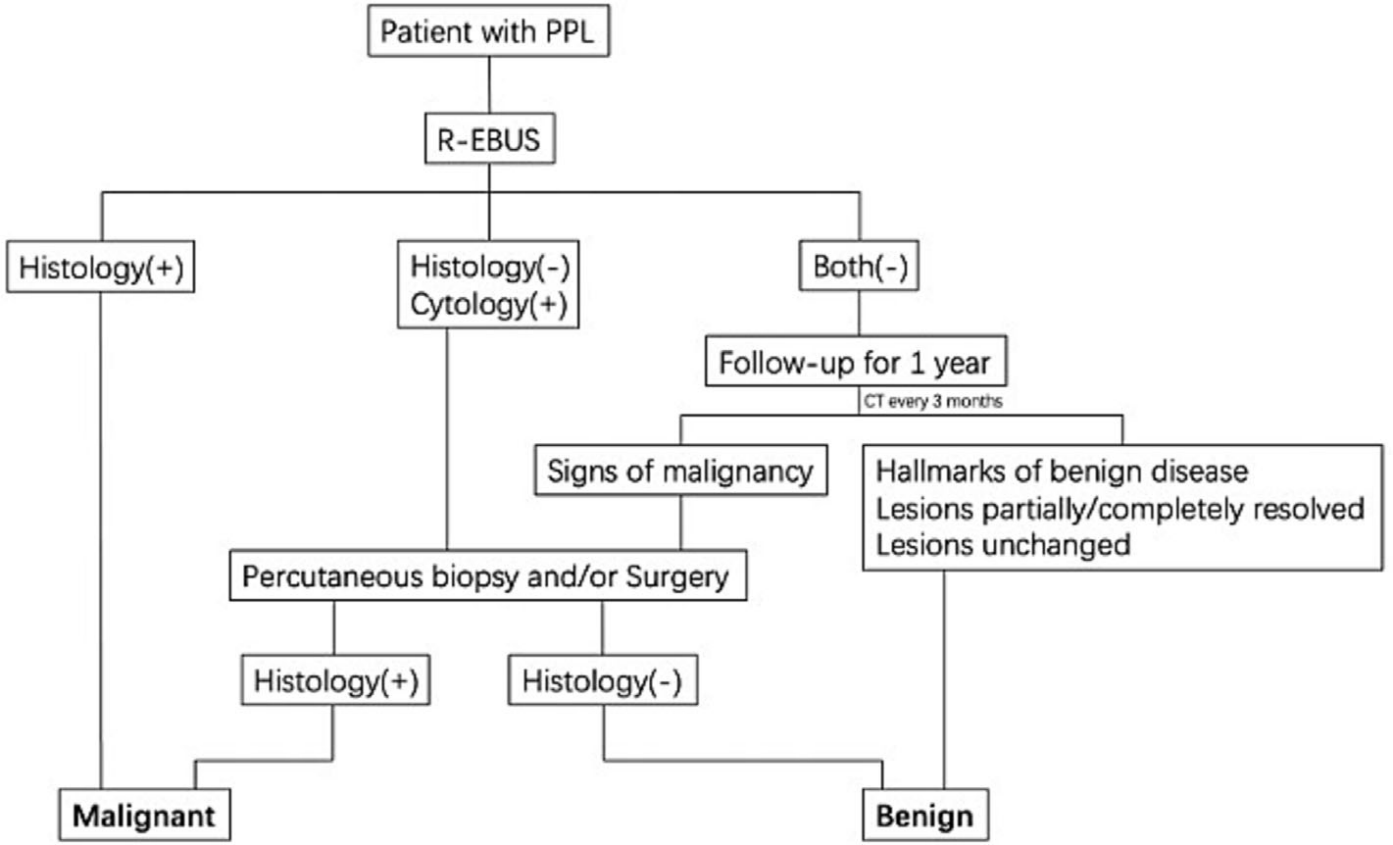
Diagnostic workflow.

Clinical information, chest CT manifestations (including size and distance between PPL and pulmonary hilum or chest wall), and features under bronchoscopy (whether the probe was within at the center of the lesion or adjacent to it) were recorded. Final diagnosis, malignant or benign, was based on pathological study and follow‐up over 1 year (Figure 1): Malignancy was confirmed by histopathology from bronchoscopy, or later by percutaneous or surgical biopsy/resection. Benign disease was confirmed with (i) definitive hallmarks for diseases such as tuberculosis, aspergillus, and noninfectious granulomatosis without evidence of malignancy during 1‐year follow‐up; (ii) there was no definitive feature of either malignancy or the above benign diseases, but the PPLs were partially or completely resolved, or unchanged throughout 1‐year follow‐up.

### 2.2 R‐EBUS‐guided sampling

Bronchoscopes with ≥2 mm biopsy channel were used (OLYMPUS BF‐P290, OLYMPUS BF‐260, and OLYMPUS BF‐1T260). R‐EBUS was performed according to routine process with virtual navigation planner and cone‐beam CT as needed.^16^ R‐EBUS probe (UM‐S20‐17S, Olympus, Tokyo, Japan) with guide‐sheath was introduced into the biopsy channel and advanced to the PPL with or without navigation. Upon lesion confirmation by EBUS or cone‐beam CT, the probe was withdrawn from the guide‐sheath, which remained in the lesion. Forceps and brush were inserted into the sheath to reach the lesion, and four to six biopsies and two brushings were performed for pathological and cytological study, and bronchial washing/lavage with 40–100 ml of normal saline instilled to the target bronchial segment was performed for cytological study.

Diagnosis made by R‐EBUS was based on histopathological findings via R‐EBUS‐guided transbronchial biopsies and/or cytological findings via brush and bronchial washings. Samples were interpreted by two pathologists at the Pathology Department of Peking University First Hospital, who were blinded to the patients' clinical information and DNA‐ICM results.

### 2.3 DNA‐ICM

Ten milliliter of bronchial washings were successively centrifuged, stained, and fixed, according to methods previously reported.^13^ A fully automated, high‐resolution image cytometer (DNA Ploidy Analysis System from Landing Medical Hi‐Tech Co., Ltd. Wuhan, China) was used to measure nuclei characteristics and DNA distribution.^17^ The DNA content was tested by Feulgen staining of the nuclei and proportionally indicated by color intensity. The nuclei of Feulgen‐stained cells were automatically scanned by an optical automated 3D moving platform, and the DNA index (DI) or DNA content (c) was calculated. Quantitative analysis of cellular DNA was performed according to the DNA Quantification System diagnostic software. Aneuploid cells were defined as cells with DNA content (c) >5(c). DNA‐ICM positive was defined as >1 aneuploid cell. This threshold is based on results described in Section 3.2, as determined by receiver operating characteristic (ROC). The aneuploid cell count was recorded, as well as the highest value of DNA content in a single aneuploid cell.

### 2.4 Statistical analysis

The data were analyzed by SPSS 19.0 software. Binary logistic regression analysis was used to analyze factors associated with positivity rate. The sensitivity, specificity, positive predictive value, and negative predictive value for different methods were calculated. ROC curve analysis was used to establish the optimal cutoff value for max value of DNA content and aneuploidy cell counts in DNA‐ICM, which have not previously been evaluated. *P* value <0.05 was considered statistically significant.

## 3 RESULTS

### 3.1 Clinical information

One hundred twelve patients were enrolled and signed the informed consent. Eleven patients were excluded from data analysis. Among them, two had visible lesions on bronchoscopy, one patient was lost to follow‐up, and two had no lesions detected by radial EBUS. Another two were excluded for abundant purulent secretion on bronchoscopy because of high false positivity of DNA‐ICM in infection.^13^ Four patients were not included with <500 cells in the sample for DNA‐ICM. Ultimately, 101 patients were included in this study. There were 54 males and 47 females with an average age of 61 ± 11 years. No severe complications, such as massive hemorrhage or pneumothorax, that required management were observed.

As shown in Table 1, there were 64 (63.4%) patients with malignant tumors, of whom 33 were diagnosed by R‐EBUS‐guided transbronchial biopsies and/or bronchial brush and wash cytology, and 31 were confirmed later by surgery/percutaneous biopsy. Baseline radiologic features of the malignant PPLs are shown in Table 2, including lesion size (indicated by max diameter), location, distance between the center of the lesion and the hilum, distance between the center of the lesion and the chest wall, as measured on CT, and whether the ultrasound probe was located at the center of the lesion or adjacent to the lesion during R‐EBUS. The majority of malignant PPLs were lung adenocarcinoma, followed by squamous carcinoma, metastatic carcinoma, small cell lung cancer and other types. The remaining 37 patients were finally diagnosed with benign lesions, based on clinical information and 1‐year follow‐up data. Of them, six were with tuberculosis infection, two with aspergillosis, and 22 with lesions partially/completely dissolved during follow‐up. Seven patients had lesions unchanged for over 1 year.

**TABLE 1:** Demographic characteristics.

	Malignant (*N* = 64)		Benign (*N* = 37)	
Male (%)	34 (53.12)		20 (54.05)	
Age median (range)	57 (20–78)		63 (34–82)	
No. of smokers (%)	30 (46.88)		18 (48.65)	
Final diagnosis (%)	ADC	44 (68.75)	Tuberculosis	6 (16.22)
	SQC	11 (17.19)	Aspergillus	2 (5.40)
	SCLC	2 (3.13)	Lesion shrinkage	22 (59.46)
	Metastasis^a^	3 (4.68)	Lesion stable	7 (18.92)
	Others^b^	4 (6.25)		

Abbreviations: ADC, adenocarcinoma; SCLC, small cell lung cancer; SQC, squamous carcinoma.

^a^ Pulmonary metastasis, included one cervical cancer; one bladder cancer; one renal cancer.

^b^ One pulmonary lymphoma; one carcinoid; two salivary gland tumors.

**TABLE 2:** Baseline radiologic features of malignant lesions.

	*N* = 64(%)
Max diameter (cm)	
0 < 2	9 (14.1)
2–4	36 (56.2)
≥4	19 (29.7)
Location	
Upper lobe	29 (45.3)
Nonupper lobe	35 (54.7)
Length to hilum (cm)	
<3	10 (15.6)
3–6	34 (53.1)
≥6	20 (31.3)
Length to chest wall (cm)	
<1	21 (32.8)
1–3	33 (51.6)
≥3 cm	10 (15.6)
Ultrasound probe	
Center	56 (87.5)
Adjacent	8 (12.5)

### 3.2 Optimal cutoff value for DNA‐ICM

**FIGURE 2:**
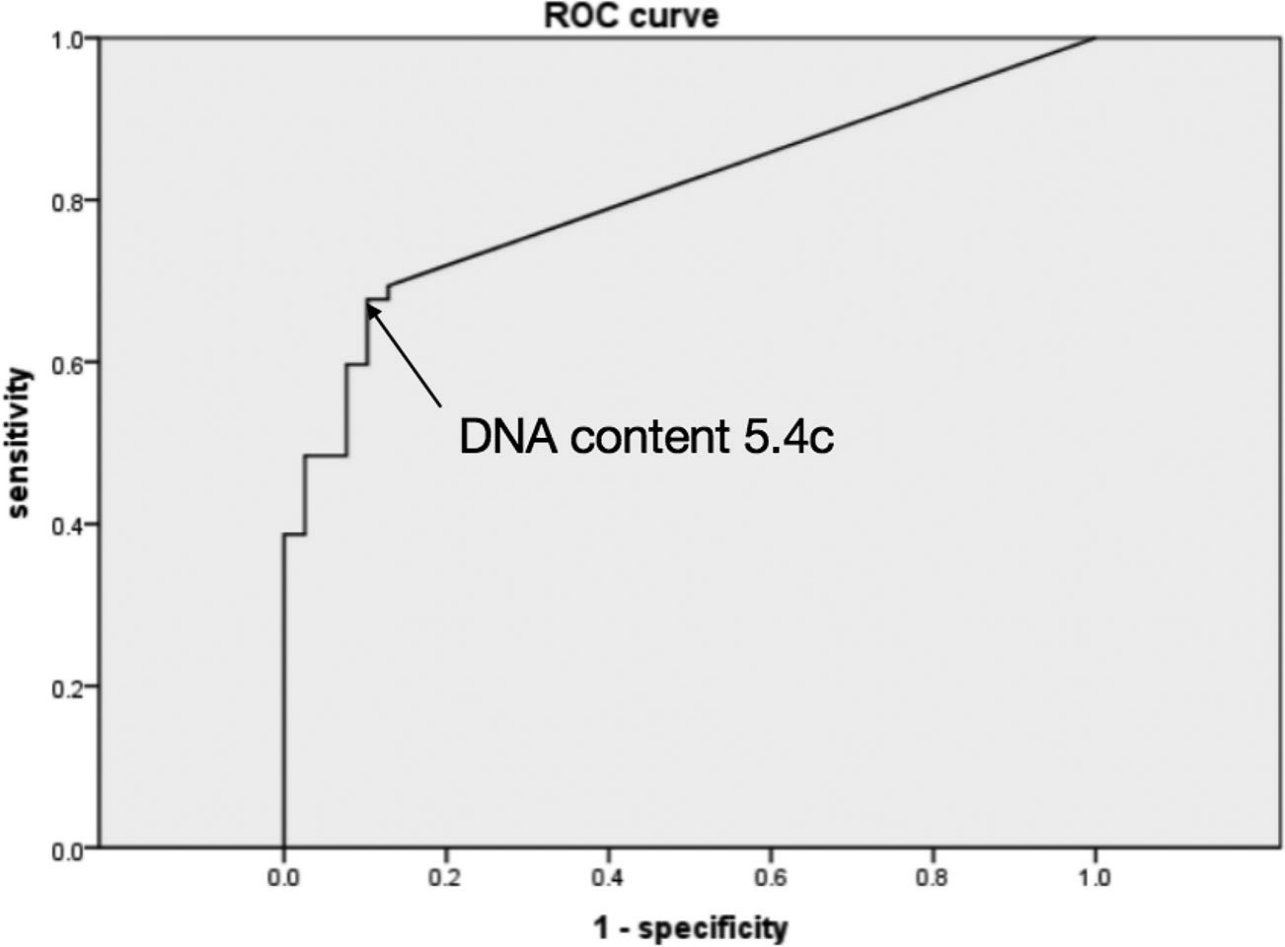
Receiver operating characteristic (ROC) curve of max DNA content (c) for diagnosis of malignant peripheral lung lesions (PPLs).

**FIGURE 3:**
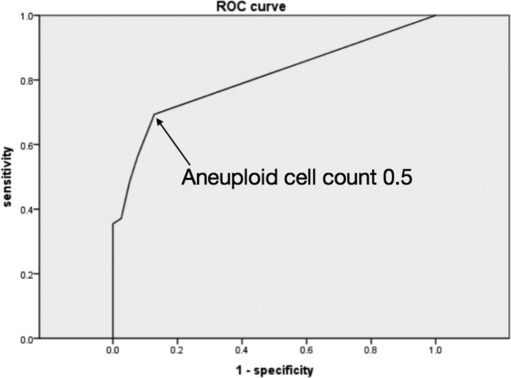
Receiver operating characteristic (ROC) curve of aneuploid cell count for diagnosis of malignant peripheral lung lesions (PPLs).

To determine the optimal cutoff value for DNA‐ICM in lung cancer diagnosis, which has not been specified in previous literatures, ROC curve analysis was performed respectively for maximal DNA value and aneuploid cell counts as predictors of malignant PPLs. For maximal DNA value (Figure 2), the area under curve was 0.806 (95% CI 0.722–0.890, *P* < 0.05), and the optimal cutoff value based on the Youden Index was 5.4(c). For aneuploid cell counts (Figure 3), the area was 0.804 (95% CI 0.720–0.889, *P* < 0.05), and the optimal cutoff value was 0.5. Based on these results, 5(c) is suggested as the cutoff value of maximal DNA content while 1 for that of aneuploid cell counts (sensitivity 67.2%, specificity 86.5%, accuracy 63.4%). In this study, the above criteria were adopted.

### 3.3 Pathology and DNA‐ICM outcomes

Pathology and DNA‐ICM outcomes for the 64 patients with malignancy are shown in Table 3. Consistencies and discrepancies between DNA‐ICM results and pathology (including histopathology and cytology) are shown. Consistencies with cytology results are also shown separately. DNA‐ICM and pathology results were consistent in 62.5% (40/64) of malignant patients; DNA‐ICM and cytology results were consistent in 32% (32/64) of patients. In 17(26.6%) malignant patients, DNA‐ICM was positive while pathology results were negative. Twenty‐nine patients had positive DNA‐ICM but negative cytology.

**TABLE 3:** Consistencies between DNA‐ICM and pathology or cytology yields.

	*Pathology (histopathology and cytology) N* = 64(%)		*Cytology only N* = 64(%)
DNA‐ICM (+) and pathology (−)^a^	17 (26.6)	DNA‐ICM (+) and cytology (−)	29 (45.3)
DNA‐ICM (−) and pathology (+)^b^	7 (10.9)	DNA‐ICM (−) and cytology (+)	3 (4.7)
Both (+)	26 (40.6)	Both (+)	14 (21.9)
Both (−)	14 (21.9)	Both (−)	18 (28.1)

^a^ Pathology (−): R‐EBUS guided biopsy (histopathology) and bronchial brush/washing (cytology) were both negative for malignant cells.

^b^ Pathology (+): Histopathology and/or cytology were positive for malignant cells.

As for the 37 patients with benign lesions, five (13.5%) were positive for DNA‐ICM but pathologically negative. Of them, two had three aneuploid cells and one patient had maximal DNA content equal or more than 7(c). The lung lesions of three patients resolved completely during follow‐up. One patient was confirmed with tuberculosis infection, and most of the lesion was dissolved after antituberculosis therapy. Another one's lesion became smaller during follow‐up. The other 32 patients (86.5%) were negative for both DNA‐ICM and pathology.

### 3.4 Positivity rate of R‐EBUS combined with DNA‐ICM

Table 4 shows the diagnostic value of R‐EBUS‐guided pathology, DNA‐ICM and the combination for PPLs. The specificity of R‐EBUS‐guided pathology was 100%, while sensitivity was low at 51.6%. Its positive predictive value (PPV) was 100%, and the negative predictive value (NPV) was 54.4%. DNA‐ICM sensitivity was 67.2%, which was slightly higher than R‐EBUS‐guided biopsy. Its specificity was 86.5%, acceptably lower. The PPV and NPV were 89.6% and 60.4%, respectively. When the two methods were combined, the sensitivity increased to 78.1% with a specificity of 86.5%. The PPV and NPV were 90.9% and 69.6% respectively.

**TABLE 4:** Diagnostic value of different methods for malignant PLLs.

	Sensitivity (%) (95% CI)	Specificity (%) (95% CI)	PPV (%) (95% CI)	NPV (%) (95% CI)
Pathology	51.6 (38.73–64.25)	100 (89.42–100)	100 (89.42 ~ 100)	54.4 (41.88 ~ 66.55)
DNA‐ICM	67.2 (54.31–78.41)	86.5 (71.23–95.46)	89.6 (77.34 ~ 96.53)	60.4 (46.00–73.55)
Combination	78.1 (66.03–87.49)	86.5 (71.23–95.46)	90.9 (80.05–96.98)	69.6 (54.25 ~ 82.26)

Abbreviations: NPV, negative predictive value; PPV, positive predictive value.

Furthermore, we assessed factors that might affect the positivity rate of pathology and its combination with DNA‐ICM. Table S1 shows the distribution of pathology and DNA‐ICM results in regard to radiologic and histological factors. Univariate analysis by binary logistic regression was then performed to determine influencing factors of positivity rate (Table 5). As for diagnosis based on R‐EBUS‐guided pathology, the positivity rate was significantly higher when the probe was located in the center of the lesion as opposed to adjacent to it (OR 9.333[95% CI, 1.075–81.020], *P* = 0.043). This was in accordant with the combination method (OR 49.000[95% CI, 5.217–460.254], *P* = 0.001). Regarding the maximal diameter of the lesion, there was no effect on the positivity rate of pathology alone, while for the combination method, lesion size was positively associated with diagnostic yield (OR 2.639[95% CI, 1.308–5.326], *P* = 0.007). The factors “distance to hilum,” “distance to chest wall,” “lesion location (upper lobe versus non‐upper lobe),” and “histopathologic type (adenocarcinoma versus non‐adenocarcinoma)” were not significantly associated with diagnostic yield in either method.

**TABLE 5:** Univariate analysis of influencing factors for diagnostic yield in malignant lesions (*N* = 64).

Factor	Malignant PPL diagnosed by R‐EBUS		Malignant PPL diagnosed by R‐EBUS combined with DNA‐ICM	
	OR (95% CI)	*P*	OR (95% CI)	*P*
Max diameter (cm)	1.338 (0.977, 1.833)	0.070	2.639 (1.308, 5.326)	0.007*
Distance to hilum (cm)	0.547 (0.254,1.181)	0.124	0.545 (0.212, 1.400)	0.208
Distance to chest wall (cm)	0.908 (0.651, 1.267)	0.570	1.046 (0.696, 1.573)	0.828
Upper lobe (*N* = 29)	1.682 (0.623, 4.546)	0.305	0.786 (0.240, 2.575)	0.691
Probe located in center of the lesion (*N* = 56)	9.333 (1.075, 81.020)	0.043*	49.000 (5.217, 460.254)	0.001*
ADC (*N* = 44)	0.373(0.124, 1.118)	0.078	0.529 (0.130, 2.155)	0.375

Abbreviation: ADC, adenocarcinoma.

* *P* < 0.05.

## 4 DISCUSSION

Early diagnosis is a key factor in reducing lung cancer mortality. Precise diagnosis of PPLs with low complications remains a challenge although various methods have been developed.^18^ The present study is the first prospective one on evaluating the sensitivity and specificity of combining R‐EBUS and DNA‐ICM in the diagnosis of malignant peripheral pulmonary lesions. Combined R‐EBUS‐guided sampling and DNA‐ICM showed a higher sensitivity of 78.1% and a specificity of 86.5%. Sensitivity of either R‐EBUS‐guided sampling or DNA‐ICM alone was 51.6% and 67.2% respectively. The sensitivity of R‐EBUS for malignant PPLs in this study was lower than previous reports.^2^, ^7^, ^9^ One possible reason is that there were eight patients where the ultrasound probe was located adjacent to rather than central to the lesion, and seven of them yielded false negative results, which could have contributed to low sensitivity. The sensitivity and specificity of the combined methods imply that DNA aneuploidy analysis could be a useful addition to R‐EBUS‐guided sampling of PPLs.

Currently, there are no consensus recommendations on the cutoff value of DNA content or aneuploid cell counts. Although maximal DNA content >5(c) is widely accepted, it has also been suggested that maximal DNA content >9(c) has higher diagnostic accuracy^19^, ^20^ with specificity almost 100%, though lower sensitivity.^21^ As for the threshold for aneuploid cells, at least one aneuploid cell is frequently used to predict malignancy. If the number is increased, the sensitivity will drop and the specificity will increase.^22^, ^23^ Through ROC curve analysis, we identified the optimal cutoff value for DNA content and aneuploid cell counts based on the Youden Index and determined more than one cell with DNA content >5(c) to be the optimal threshold. These cutoff values were used in the subsequent analyses.

In this study, five benign patients had positive DNA‐ICM results. Reasons for this may be as follows: (1) Aneuploid cells may also be present in non‐neoplastic diseases such as infection. High false positive rate has been shown in such patients,^13^ which was the reason for exclusion of patients with clinical manifestations of infection or purulent secretions under bronchoscopy. Of the enrolled patients, one patient with confirmed tuberculosis had positive DNA‐ICM. The false positivity had also been reported in a previous study.^14^ (2) Aneuploid cells have been reported in normal tissues and can lead to false positive DNA‐ICM. However, this phenomenon is relatively rarer in the lung.^24^ (3) Cells with DNA content of more than 5(c) can also be observed in some benign lung diseases.^25^, ^26^ One of the mechanisms could be cellular damage induced by oxygen radicals or inflammation which can occur in various diseases. As in this study, there were three patients with positive DNA‐ICM, yet their lesions dissolved completely during follow‐up. (4) In cervical cancer studies, it might take several years from the detection of aneuploid cells to the confirmation of cancer.^27^ Among the benign lesions with positive DNA‐ICM, one patient's lesion became smaller after 2 years of follow‐up, indicating that longer periods of follow‐up are needed in such cases.

Seven patients had false negativity of DNA‐ICM. Some malignant tumors may only contain diploid DNA, which can lead to false negative DNA‐ICM results. DNA diploid tumors have been found in over half of hematologic tumors, and also in solid tumors.^28^ A meta‐analysis reported that the prevalence of aneuploidy in NSCLC was 65%.^29^ Thus, the false negativity could be due to tumor DNA diploidy.

It is also worth mentioning that PPLs, with negative pathology but positive DNA‐ICM, should be closely followed in case of progression to an advanced stage. Repeat biopsy such as CT‐guided percutaneous lung biopsy or surgery may be warranted. Of the 22 patients with positive DNA‐ICM but negative pathology, 17 (77.3%) were eventually diagnosed with malignant lesions, while five (22.7%) were diagnosed as benign lesions. If pathology and DNA‐ICM are both negative, especially when the probe was in the lesion, there is a high negative predictive value that may allow this subset of patients to avoid futile invasive procedures, particularly thoracic surgery.

Age, sex, smoking status, CT manifestations, and location of the probe were analyzed to determine factors influencing diagnostic yield. Lesions in the upper lobe typically have a higher risk of malignancy,^8^, ^30^ but no correlation was found between location and diagnostic yield. Lesion size, though no significance in R‐EBUS yield, was positively associated with positivity rate of R‐EBUS combined with DNA‐ICM. An earlier study using EBUS with guide‐sheath for PPLs demonstrated similar diagnostic efficacy in lesions of different sizes (≤10 mm, 10–15 mm, 15–20 mm, 20–30 mm, >30 mm).^31^ Another study also found that PPLs >20 mm had significantly greater diagnostic yield than <20 mm.^32^ In this study, DNA‐ICM was perhaps influenced more by lesion size than R‐EBUS. The location of the probe had more significant effect on yield than lesion size. Fourteen patients diagnosed with cancer had both negative DNA‐ICM and pathology, seven of which the ultrasound probe was located adjacent to rather than inside the lesion. When the probe was inside the lesion, the yield was significantly higher, consistent with several other studies,^7^, ^8^, ^33^, ^34^, ^35^ and the probable reason for the false negative R‐EBUS‐guided sampling and DNA‐ICM results in this study. Distance between the lesion and the hilum or chest wall were not significant influencing factors.

The study had the following limitations: (i) Due to the COVID‐19 pandemic, some patients did not strictly adhere to follow‐up, and follow‐up CT was delayed for months in some patients. However, all the patients had confirmed final diagnosis at the end of the study. Only one patient was lost to follow‐up, which had minimal influence on the results. (ii) The false negative rate of the combination for malignant lesions was high at 21.9%. Nevertheless, when excluding patients where the probe was adjacent to the lesion, the false negative rate decreased to an acceptable level of 10.9%. (iii) The false positive rate of DNA‐ICM was 13.5%. Apart from assisting in confirming diagnosis of malignancy, another core value of utilizing DNA‐ICM for the diagnosis of PPLs could be avoiding a delay in diagnosis of patients who do indeed have a lung neoplasm.

## 5 CONCLUSIONS

This study found that DNA‐ICM was a useful additional tool to R‐EBUS when diagnosing peripheral lesions. DNA‐ICM combined with R‐EBUS‐guided biopsy/bronchial brush and wash cytology improved the sensitivity and could help distinguish patients who, despite a negative R‐EBUS pathology result, require further biopsy to confirm malignancy. An optimal cutoff value for DNA‐ICM was also established in this prospective study, which could help promote a future consensus in the use of DNA‐ICM in assisting cancer diagnosis. DNA‐ICM is easy to perform and interpret, and its combined use with radial‐EBUS in the diagnosis of PPLs is strongly recommended.

## AUTHOR CONTRIBUTIONS

**Cuiyan Guo:** Conceptualization; methodology; investigation; data analysis; writing (original draft). **Qi Zhang:** Investigation; methodology; resources; writing (editing). **Yan Hu:** Methodology; investigation; data collection and analysis. **Junfang Huang:** Investigation; data collection and analysis. **Shixuan Wang:** Investigation; data collection and analysis. **Guangfa Wang:** Conceptualization; methodology; supervision; writing (review and editing).

## CONFLICT OF INTEREST STATEMENT

The authors declare no conflict of interest.

## ETHICS STATEMENT

The study was conducted in accordance with the Declaration of Helsinki, and approved by the Ethics Committee of Peking University First Hospital (2019‐072).

## INFORMED CONSENT STATEMENT

Written informed consent has been obtained from the patient(s) to publish this paper.

## Supporting information


**Data S1** Supporting Information.

## Data Availability

The data that support the findings of this study are available from the corresponding author upon reasonable request.
