# Supplementary material for: DNA image cytometry of bronchial washing as a diagnostic adjunct to radial endobronchial ultrasound‐guided sampling of peripheral lung lesions: A single center prospective study
Source: Clin Respir J. 2023 Dec 11;18(1):e13703. doi: 10.1111/crj.13703 (PMC10775888; doi:10.1111/crj.13703)
Supplement: Supplementary file 1 — Data S1 Supporting Information. [file CRJ-18-e13703-s001.docx]

**Supplementary table 1**

Distribution of pathology and DNA-ICM results according to radiologic and histologic factors

| Factor | Only pathology(+)  N=7 | Only DNA-ICM(+)  N=17 | Both positive  N=26 | Both negative  N=14 |
| --- | --- | --- | --- | --- |
| Max diameter(cm) |  |  |  |  |
| 0<2 (N=9) | 1 | 5 | 1 | 2 |
| 2-3 (N=19) | 3 | 1 | 4 | 11 |
| 3-4 (N=17) | 3 | 4 | 9 | 1 |
| ≥4(N=19) | 0 | 7 | 12 | 0 |
| Location |  |  |  |  |
| Upper lobe（N=29） | 3 | 5 | 14 | 7 |
| Non-upper lobe（N=35） | 4 | 12 | 12 | 7 |
| Distance to hilum (cm) |  |  |  |  |
| <3(N=10) | 0 | 2 | 7 | 1 |
| 3-6(N=34) | 4 | 9 | 14 | 7 |
| ≥6(N=20) | 3 | 6 | 5 | 6 |
| Distance to chest wall(cm) |  |  |  |  |
| <1(N=21) | 0 | 6 | 7 | 5 |
| 1-3(N=33) | 3 | 6 | 16 | 7 |
| ≥3cm(N=10) | 4 | 5 | 3 | 2 |
| Ultrasound probe |  |  |  |  |
| Center (N=56) | 7 | 17 | 25 | 7 |
| Adjacent (N=8) | 0 | 0 | 1 | 7 |
| Pathology |  |  |  |  |
| ADC(N=44) | 6 | 7 | 20 | 11 |
| SCC(N=11) | 0 | 5 | 5 | 1 |
| SCLC(N=2) | 0 | 2 | 0 | 0 |
| Metastasis(N=3) | 0 | 2 | 1 | 0 |
| Other(N=4) | 1 | 1 | 0 | 2 |

ADC, adenocarcinoma; SCC, squamous cell carcinoma; SCLC, small cell lung cancer.
